# Biocontrol and Rapid Detection of Food-Borne Pathogens Using Bacteriophages and Endolysins

**DOI:** 10.3389/fmicb.2016.00474

**Published:** 2016-04-08

**Authors:** Jaewoo Bai, You-Tae Kim, Sangryeol Ryu, Ju-Hoon Lee

**Affiliations:** ^1^Department of Food and Animal Biotechnology, Department of Agricultural Biotechnology, Center for Food and Bioconvergence, Research Institute of Agriculture and Life Sciences, Seoul National UniversitySeoul, South Korea; ^2^Department of Food Science and Biotechnology and Institute of Life Science and Resources, Kyung Hee UniversityYongin, South Korea; ^3^Institute of Food Industrialization, Institutes of Green Bio Science and Technology, Seoul National UniversityPyeongchang, South Korea

**Keywords:** bacteriophage, food-borne pathogens, biocontrol, rapid detection, endolysin, cell-wall binding domain, reporter phage, food preservatives

## Abstract

Bacteriophages have been suggested as natural food preservatives as well as rapid detection materials for food-borne pathogens in various foods. Since *Listeria* monocytogenes-targeting phage cocktail (ListShield) was approved for applications in foods, numerous phages have been screened and experimentally characterized for phage applications in foods. A single phage and phage cocktail treatments to various foods contaminated with food-borne pathogens including *E. coli* O157:H7, *Salmonella enterica, Campylobacter jejuni, Listeria monocytogenes, Staphylococcus aureus, Cronobacter sakazakii*, and *Vibrio* spp. revealed that they have great potential to control various food-borne pathogens and may be alternative for conventional food preservatives. In addition, phage-derived endolysins with high host specificity and host lysis activities may be preferred to food applications rather than phages. For rapid detection of food-borne pathogens, cell-wall binding domains (CBDs) from endolysins have been suggested due to their high host-specific binding. Fluorescence-tagged CBDs have been successfully evaluated and suggested to be alternative materials of expensive antibodies for various detection applications. Most recently, reporter phage systems have been developed and tested to confirm their usability and accuracy for specific detection. These systems revealed some advantages like rapid detection of only viable pathogenic cells without interference by food components in a very short reaction time, suggesting that these systems may be suitable for monitoring of pathogens in foods. Consequently, phage is the next-generation biocontrol agent as well as rapid detection tool to confirm and even identify the food-borne pathogens present in various foods.

## Introduction

Food safety is one of the major concerns due to threatening human health by various food-borne pathogens. Every year in the United States, about 9.4 million cases of foodborne illness with about 56,000 hospitalizations and 1,300 deaths caused by major food-borne pathogens including *Salmonella, Clostridium perfringens, Listeria monocytogenes*, and *Campylobacter* have been reported (Scallan et al., 2011). Because of food contaminations by pathogens, about 25% of their food productions were lost in food industries every year (Sarhan and Azzazy, 2015). In general, control of these food-borne pathogens has been done using various natural or chemical food preservatives. Natural preservatives such as organic acids, bacteriocins, chitosan, and lactoferrin have tendency to exhibit weak and limited antimicrobial activities (Juneja et al., 2012). However, consumers generally do not prefer chemical preservatives due to their known side effects (Pawlowska et al., 2012). Furthermore, while antibiotics have strong and stable antimicrobial activities, they are not allowed for applications in foods.

Bacteriophages are bacterial viruses with host specificity and lysis activities, indicating that they can infect and lyse the specific host bacteria for their replication and propagation (Kutter and Sulakvelidze, 2005). Therefore, bacteriophages have been suggested as natural biocontrol agents against food-borne pathogens without any harm to human cells, indicating their safety (McCallin et al., 2013). In general, phages containing double-stranded DNA genomes have specific host cell wall lysis enzymes called endolysin for bacterial host lysis (Borysowski et al., 2006). This enzyme has two protein domains, peptidoglycan-hydrolyzing enzymatic activity domain (EAD) for host cell lysis and cell wall binding domain (CBD) for specific host recognition (Nelson et al., 2012). In general, endolysin is externally added to lyse gram-positive bacteriaand the related animal study showed no side effect, suggesting that it should be safe for human (Jado et al., 2003; Yang et al., 2012; Jun et al., 2014b). Due to these distinct characteristics, endolysin has been considered as a novel type natural food preservative against food-borne pathogens (Schmelcher and Loessner, 2015).

In addition to the biocontrol of food-borne pathogens in foods using phage or endolysin, their rapid detection is also important in the prevention of food-borne outbreaks (Hagens and Loessner, 2007). For the rapid detection without enrichment step of food-borne pathogens, PCR- and antibody-based rapid detection methods have been developed and broadly used. However, these methods have some limitations including detection limit (antibody) and requirement of long amplification time (PCR; Yamamoto, 2002; Fratamico et al., 2005; Schmelcher and Loessner, 2014). To reduce these limitations, phage-derived CBD and genetically engineered reporter phage have been newly proposed and considered for rapid detection of food-borne pathogens in foods (Kim et al., 2014; Schmelcher and Loessner, 2014). These new rapid detection methods can overcome limitations of conventional detection methods and enhance the detection limit and sensitivity in foods (Schmelcher et al., 2010; Smartt et al., 2012). In addition, these novel rapid detection methods could be used for monitoring of pathogens in foods. Therefore, these new technologies would provide novel approaches for rapid detection of food-borne pathogens in food environments.

This review is focused on the biocontrol and rapid detection of various food-borne pathogens in foods using phages and their derivatives including endolysin, CBD, and reporter phage. Therefore, general features and various food applications of phages and endolysins for biocontrol of food-borne pathogens would be explained and discussed in this review. In addition, CBD and reporter phage would be reviewed as a novel type of rapid detection and monitoring of food-borne pathogens with most recent study cases. This review would provide novel insights into applications of phages and their derivatives for efficient biocontrol and rapid detection of various food-borne pathogens in highly complexed food environments.

## Bacteriophage biology

### General features and phylogeny

Bacteriophages are the most abundant microorganisms on Earth, and also have the ability to infect bacteria. Basic structure of phages in the order *Caudovirales* consists of two parts: phage head and its tail. The phage head contains a genetic material in a form of DNA or RNA (Clark and March, 2006). Linked to the phage head, the phage tail generally plays roles in recognition and adsorption of the specific bacterial host receptor (Bertin et al., 2011). After binding to the host bacterium, phage injects its genetic material into the host cytosol via tail structure by diffusion, osmotic pressure, or transport by specific protein (Grayson and Molineux, 2007; Ming et al., 2011). The injected genetic material undergoes host genome integration for lysogenic cycle or replication for lytic cycle. During the lytic cycle, structural proteins are produced from encoded genes in the phage genome. After replication of the genetic material and production of structural proteins, progeny phages are assembled with them and released from the host bacterium (Inal, 2003; Kutter and Sulakvelidze, 2005).

Since the first bacteriophage was discovered and characterized by d'Hérelle (1917) and Duckworth (1976), tailed bacteriophages in the order *Caudovirales* are the most abundant (about 96% of all phages). This order consists of three major families including *Siphoviridae, Myoviridae* and *Podoviridae* with different morphological characteristics. Among the reported phages to date, phages in the *Siphoviridae* family are the most abundant (61.6% of all phages) with long flexible non-contractile tails ranged from 79 to 539 nm. The phages in the *Myoviridae* family are the second most abundant (24.5%) and they have larger heads ranged from 53 to 160 nm in comparison to those of other two families. Moreover, contractile tails give *Myoviridae* its unique characteristics. The phages in the *Podoviridae* family (13.9%) have a distinctly short non-contractile tail ranged from 3 to 40 nm (Ackermann, 1998).

### Phage therapy

The first clinical study using phages was a direct phage injection in six patients with staphylococcal boils in 1921 (Deresinski, 2009). Since then, phages have been used to cure various diseases caused by bacterial infections for several decades in Eastern Europe. However, antibiotics have been widely used for the same purpose in other parts of the world and this resulted in the emergence of antibiotic-resistant bacteria. Therefore, it has been big issue how to control these antibiotic-resistant bacteria. Because phage has recently attracted the public attention due to its high host specificity and efficient host lysis, phage therapy has been revisited to control these problematic bacteria in Western Europe (Clark and March, 2006).

To date, numerous clinical phage trials have been reported against various pathogens including *E. coli, Klebsiella pneumoniae, Staphylococcus aureus, Pseudomonas aeruginosa*, and *Salmonella* Typhimurium. As an example, in Poland, 550 patients with gastrointestinal, head, neck, and skin infections caused by these pathogens were successfully treated and symptoms of 506 patients (92%) were relieved (Inal, 2003). In addition, in Russia, 1,340 patients with conjunctivitis, dermatitis, pharyngitis, and rhinitis were divided into three groups for different treatment regimens: phage treatment (360 patients), antibiotics treatment (404 patients), and combination (576 patients; Inal, 2003). Interestingly, the phage-treated and the combination groups were clinically improved up to 86 and 83%, respectively. However, the antibiotics-treated group showed minor improvement up to 48%, suggesting that phage therapy may be effective to control these pathogens but combination of phages and antibiotics did not show synergistic effect. In Ireland, 10 methicillin-resistant *S. aureus* (MRSA) DPC5246-infected human hands were soaked in a solution containing 10^8^ PFU/ml of a single phage K, revealing 2 log reduction, suggesting that MRSA can be controlled using a specific phage (O'flaherty et al., 2005c; Li et al., 2010). Based on the clinical studies, many commercial phage therapy products were developed and produced in Eastern Europe, including “Phagestaph” (JSC Biochimpharm, Tbilisi, Georgia), “E.coli bacteriophage” (Microgen, Moscow, Russia), and “Complex pyobacteriophage” (Microgen). In addition, other countries have many phage therapy-related companies producing commercial products: USA (Elanco Food Solutions, Gangagen Inc., Intralytix, Neurophage Pharmaceuticals, New Horizons Diagnostics, OmniLytics Inc., Phage International, Targanta Therapeutics, Viridax), UK (AmpliPhi Biosciences Corporation, Blaze Venture Technologies, BigDNA, Novolytics, Phico), Georgia (Biopharm Ltd., JSC Biochimpharm, Phage Therapy Center), Australia (Special Phage Services Pty, Ltd.), Canada (Biophage Pharma Inc.), Germany (Hexal Genentech), India (Gangagen Biotechnologies PVT Ltd.), Ireland (Phage Works), Israel (Phage Biotech Ltd.), Portugal (Innophage), South Korea (CJ CheilJedang Corporation), and the Netherlands (EBI Food Safety; Endersen et al., 2014). Consequently, phage therapy would provide novel insights and approaches to overcome the limitations of antibiotics and biocontrol of various antibiotics-resistant bacteria without any side effect in humans.

### Food applications

In addition to the phage therapy, phages can be used for biocontrol of various food-borne pathogens. The advantage of phage applications in foods is efficient inhibition of food-borne pathogens as well as no harm to human. In addition, antibiotics are not allowed for food applications. Therefore, food application using phages could be a good alternative approach for biocontrol of food-borne pathogens in foods. ListShield (Intralytix, Inc., Baltimore, MD, USA), a cocktail of six phages targeting *L. monocytogenes*, was first approved by the United States Food and Drug Administration (FDA) and U.S. Department of Agriculture (USDA)'s Food Safety and Inspection Service (FSIS) for applications in foods in 2006 and re-approved as GRAS status by FDA in 2014. In addition, EcoShield (Intralytix), a cocktail of three phages (ECP-100) targeting *E. coli* O157:H7, was also approved by FDA and FSIS for food applications in 2011. Listex P100 (Micreos Food Safety, Wageningen, The Netherlands), a single phage targeting *L. monocytogenes*, was approved as GRAS status by FDA in 2006. Recently, SalmoFresh (Intralytix), a cocktail of six *Salmonella*-targeting phage, was also approved as GRAS status by FDA in 2013 (Sharma, 2013). Therefore, these phage products are allowed to use in foods as food preservatives to control specific food-borne pathogens. In addition to direct food applications of phages, they can be used to prevent cross contamination of pathogens in food-contact materials as well as food processing facilities (Sulakvelidze, 2013). Furthermore, phages can be used to sanitize human hands and utensils. Therefore, phage applications would be useful for extension of food preservation periods and food safety.

## Biocontrol of food-borne pathogens using phages and endolysins

### Phage applications

As discussed previously, phages can control food-borne pathogens by host recognition, infection, and lysis. In this section, phage applications would be explained and discussed with various research reports of each host pathogenic bacterium. Various phage applications in foods are summarized and listed in Table 1.

**Table 1:** **Examples of phages used in studies related to pathogen reduction in foods**.

**Host**	**Phage**	**Applications**	**Results**	**References**
*E.coli* O157:H7	FAHEc1	Thinly sliced beef pieces	2.7 log reduction occurred with 3.2 × 10^7^ PFU/4 cm^2^ treatment	Hudson et al., 2013
	DT1, DT6 (phage cocktail)	Cow meat	2.2 log reductions at 5°C after 24 h	Tomat et al., 2013b
	DT1, DT6 (phage cocktail)	Milk fermentation	3.0 log reduction (total inactivation)	Tomat et al., 2013a
	ECP100 (3 phages cocktail)	Hard surfaces	Reduction of 99.99, 98, and 94% of viable cell number with 10^10^, 10^9^, and 10^8^ PFU/ml treatment, respectively	Abuladze et al., 2008
	ECP100 (3 phage cocktail)	Tomato slice, spinich, ground beef	(Tomato slice) reduction of 99, 94, and 96% of viable cell number during storage at 10°C for 24 h, 120 h, and 168 h, respectively (spinich) 100% reduction of viable cell number during storage at 10°C for 24 h and 120 h 99% reduction of viable cell number during storage at 10°C for 168 h (ground beef) 95% reduction of viable cell number during storage at 10°C for 24 h	Abuladze et al., 2008
	e11/2, e4/1c	*Ex vivo* rumen model	(Phage e11/2) reduction below the detection limit within 1 h, (Phage e4/1c) reduction of bacterial cell numbers within 2 h	Rivas et al., 2010
	e11/2, e4/1c (phage cocktail)	Hide samples	2.02 log CFU/cm^2^ reduction after 1 h	Coffey et al., 2011
	e11/2, e4/1c, PP01 (phage cocktail)	Beef	Reduction of *E. coli* O157:H7 counts less than 10 CFU/ml at 37°C after 1 h	O'Flynn et al., 2004
	BEC8	Leafy green vegetables	Reduction of 10^6^ CFU/ml of *E. coli* O157:H7 with simultaneous treatment of BEC8 (approx. 10^6^ PFU/leaf), and trans-cinnamaldehyde (TC) (0.5% v/v) at both 23°C and 27°C after 24 h	Viazis et al., 2011b
	BEC8	Materials typically used in food processing surfaces	More than 3 log reduction at temperatures above 12°C within 10 min on all 3 surfaces	Viazis et al., 2011a
*Salmonella enterica*	F01-E2	Hot dogs, cooked and sliced turkey breast, mixed seafood, chocolate milk and egg yolk	No viable bacteria were detected after treatment at 8°C during storage 2–5 log suppression of *Salmonella* growth was suppressed at 15°C during storage	Guenther et al., 2012
	wksl3	Chicken skin	2.5 log reduction in the number of bacteria from day 2 to day 7	Kang et al., 2013
	PhageA, PhageB (phage cocktail)	Broccoli seeds	1.5 log suppression of *Salmonella* growth	Dennis et al., 2010
	PA13076, PC2184 (phage cocktail)	Chicken breast, pasteurized whole milk and Chinese cabbage	(In all tested foods) reduction of 1.5–4 log CFU/sample	Bao et al., 2015
	UAB_Phi20, UAB_Phi78, UAB_Phi87	Pig skin, chicken breasts, packaged lettuce, Fresh egg	(Pig skin) 4 log reduction for *S*. Typhimurium and 2 log reduction for *S*. Enteritidis at 4°C for 7 days, (chicken breasts) 2.2 log reduction for *S*. Typhimurium and 0.9 log reduction for *S*. Enteritidis at 4°C for 7 days, (packaged lettuce) 3.9 log reduction for *S*. Typhimurium and 2.2 log reduction for *S*. Enteritidis at room temperature for 60 min, (fresh egg) 0.9 log reduction for both *S*. Typhimurium and *S*. Enteritidis	Kretzer et al., 2007; Spricigo et al., 2013
	PC1	Pig skin	Reduction of 4.1–4.3 log CFU	Hooton et al., 2011
	F1055S, F12013S	Fertile eggs	Reduction of the disease symptoms in the chicks	Giusiano et al., 2010
*Campylobacter jejuni*	phi2	Chicken skin	More than 4 log reduction at 4°C	Atterbury et al., 2003
	phiCcoIBB35, phiCcoIBB37, phiCcoIBB12 (phage cocktail)	Administration to chicken	Reduction of both *C. coli* and *C. jejuni* in feces by approximately 2 log CFU/g	Carvalho et al., 2010
	C220	Broiler chicken	2 log CFU/g reduction in cecal colonization number of *C. jejuni*	El-Shibiny et al., 2009
	CP8, CP34	Broiler chicken	0.5–5 log CFU/g reduction of cecal contents over a 5-day period	Clark and March, 2006
	NCTC12673	Chicken surface	95% reduction in the chicken portions at 4°C after 24 h	Goode et al., 2003
	Phage Cj6	Cooked and raw meat	(Cooked meat) 2.8 log reduction in bacteria number, (raw meat) 2.2 log reduction in bacteria number	Zhen et al., 2008
*Listeria monocytogenes*	A511	(Liquid foods) chocolate milk and mozzarella cheese brine (solid foods) Hot dogs, sliced turkey meat, smoked salmon, seafood, sliced cabbage, and lettuce leaves	(Liquid foods) reduction below the detection level at 6°C after 6 day, (solid foods) 5 log reduction at 6°C after 6 day	Ma et al., 2009
	A511	Soft cheese	6 log reduction for 22 days	(Guenther and Loessner, 2011)
	P100	Cheese	Complete eradication or at least 3.5 log reduction for 22 day	Kim et al., 2014
	P100	Melon slice, pear slice, apple slice	(Melon slice) 1.5 log reduction in bacteria number, (pear slice) 1.0 log reduction in bacteria number, (melon juice) 8.0 log reduction in bacteria number, (pear juice) 2.1 log reduction in bacteria number, (apple slice or juice) no significant reduction in bacteria number	Oliveira et al., 2014
	FWLLm1	Ready-to-eat chicken breast roll	2.5 log reduction and no re-growth at 5°C over 21 days	Bigot et al., 2011
	LM103, LMP-102 (Phage cocktail)	Honeydew melon, golden delicious apple	(Honeydew melon) 2.0–4.6 log reduction, (golden delicious apple) 0.4 log reduction	Devreese, 2010
	LMP-102	Honeydew melon	Reduction of viable cell number to non-detectable levels immediately after treatment and suppressed growth of the pathogen at 10°C throughout the storage period of 7 days	Leverentz et al., 2004
*Staphylococcus aureus*	IPLA35, IPLA88	Fresh type cheese, hard type cheese	(Fresh type cheese) 3.8 log CFU/g reduction in 3 h, viable cell counts were under the detection limits after 6h, (hard type cheese) 4.6 log CFU/g reduction during ripening period and only 1.2 log CFU/g of viable cell was detected at the end of ripening period	Bueno et al., 2012
	Two kinds of phage cocktails (TEAM/P68/LH1-MUT and phi812/44AHJD/phi2)	Cheddar cheese	Eradication of 10^6^ CFU/g *S. aureus* at 4°C after 14 days	El Haddad et al., 2016
*Cronobacter sakazakii*	ESP 732-1, ESP 1-3	Reconstituted infant formula	(Phage ESP 732-1) eradicating *C.sakazakii* at 12, 24, and 37°C, (phage ESP 1–3) eradicating *C. sakazakii* at 24°C	Kim et al., 2007
	CR5	Reconstituted infant formula	Reduction of 10^2^ CFU/ml with an MOI of 10^5^ of phage for 10 h	Lee et al., 2016
*Vibrio spp*.	VPp1	Oyster rearing system	2.35–2.76 log CFU/g reduction of *V. parahaemolyticus* numbers in oysters at 16°C within 36 h	Cai et al., 2011; Rong et al., 2014
	Vpms1	Brine shrimp	Effectively prevent vibriosis in brine shrimp even with and MOI of 0.45	Martínez-Díaz and Hipólito-Morales, 2013
	pVp-1	Oysters	Reduction of 3.3 log CFU/g	Jun et al., 2014a

#### *E. coli* O157:H7

*E. coli* O157:H7 belongs to the Shiga toxin producing *E. coli* (STEC), a major food-borne pathogen causing hemolytic uremic syndrome and acute renal failure with an extremely low dose (about 10^1^ cells; Kaper, 1998; Newell et al., 2010). It is generally ingested by consumption of contaminated, undercooked beef and sometimes fresh fruit juices (Cody et al., 1999; Endersen et al., 2014). Therefore, control of *E. coli* O157:H7 in foods is important for prevention of food-borne outbreaks.

*E. coli* O157:H7-targeting phages and their host inhibition activities have been reported. Phage FAHEc1 (10^7^ PFU/ml) applied to *E. coli* O157:H7 and sliced meat piece demonstrated 4 log reduction at 5°C and 2–3 log reductions at 37°C, respectively (Hudson et al., 2013). To enhance the host growth inhibition and lysis activities, phage cocktails were prepared with phages DT1 and DT6. This phage cocktail treatment showed 6.3 log reduction of *E. coli* O157:H7 and minimized the appearance of Bacteriophage Insensitive Mutants (BIMs). Application of this phage cocktail or DT6 to a beef sample at 24°C for 6 h revealed that the phage cocktail treatment (2.58 log reduction) was more effective than DT6 treatment alone (0.74 log reduction), suggesting that phage cocktail is more effective for control of food-borne pathogen than a single phage (Tomat et al., 2013b). Comparative phage experiments with a phage cocktail and a single phage DT1 with *E. coli* O157:H7-contaminated milk samples support the efficiency of the phage cocktail (Tomat et al., 2013a). Further phage cocktail experiment with eight different virulent phages (BEC8) and 123 different *E. coli* O157:H7 strains showed that >94% of the tested strains were inhibited in the host range tests (Viazis et al., 2011a), indicating that virulent phages could inhibit the growth of *E. coli* O157:H7 effectively. This suggests the effectiveness of phage treatment to control *E. coli* O157:H7 in foods. For phage applications to *E. coli* O157:H7 in foods, phage should be stable under various stress conditions including temperature, pH, water activity, and salt stress. Two different phages e11/2 and e4/1c were tested under these stress conditions, showing that they were stable under pH 4–10, -22°C to 60°C, and 1–2.5% NaCl concentration. Interestingly, phage stability test under various water activity levels showed that phage e4/1c was more stable than phage e11/2 (Coffey et al., 2011). *E. coli* O157:H7-targeting phages showed that they are highly stable for survival under various food conditions and can effectively control the pathogen in foods with a form of phage cocktail.

#### Salmonella enterica

*Salmonella* can cause disease so-called non-typhoidal salmonellosis, the most common food-borne disease with symptoms like common gastroenteritis, enteric fever and ulceration (Endersen et al., 2014). *Salmonella* has been widely detected in various animal-based foods (Plym Forshell and Wierup, 2006). However, it is very difficult to control in the food environment. Although natural and chemical food preservatives have been used for prevention of food-borne pathogen contaminations, they are not specific for *Salmonella*. As previously explained, phages have been approved as novel type food preservatives by the US FDA (Sharma, 2013) and the characteristics of phages are high host specific with lysis activities (Chang et al., 2015). Therefore, phages have been interesting subjects for biocontrol of *Salmonella* in foods (Hertwig et al., 2013).

*S*. Typhimurium-targeting phage F01-E2 was tested for food applications including hot dogs (Wiener sausages), cooked and sliced turkey breast (deli meat, cold cuts), mixed seafood (cooked and chilled cocktail of shrimps, shellfish, and squid), chocolate milk (whole milk with cocoa and sugar added), and egg yolk (pasteurized). Interestingly, this phage (3 × 10^8^ PFU/g of each food sample) was treated to *S*. Typhimurium-contaminated Ready-To-Eat (RTE) foods and then they were stored at 8°C for 6 days. After storage, no bacterial host was detected in all RTE foods, indicating that a single treatment of phage may be enough to reduce *S*. Typhimurium in RTE foods during storage even at low temperature (Guenther et al., 2012). In addition, the broad host range phage wksl3 (10^7^ PFU/ml) was treated to chicken skin contaminated with *S*. Enteritidis (10^3^ CFU/cm^2^ skin) at 8°C for 7 days, showing 2.5 log reduction (Kang et al., 2013). Its genome analysis revealed that it does not have toxin, virulence factors, food allergen-related proteins, as well as lysogen-related gene clusters, suggesting that this phage should be safe for food and human trials. Administration of high dose phage (10^11^ PFU/kg mouse body weight) to mice showed that no death or clinical pathogenicity signs were observed (Kang et al., 2013).

To enhance the host lysis activity, *Salmonella* phage cocktails were prepared and tested. A single phage and a phage cocktail of two virulent phage, PA13076 and PC2184, were applied to three different foods (chicken breast, pasteurized whole milk, and Chinese cabbage) contaminated with *S*. Enteritidis, showing that a phage cocktail treatment exhibited more effective host lysis activity (4 log reduction) than a single phage treatment (2–3 log reductions) in milk (Bao et al., 2015). Furthermore, *Samonella* phage cocktails containing three phages (UAB_Phi20, UAB_Phi78, and UAB_Phi87) and four phages (Felix01, phiSH19, phiSH17, and phiSH18) also showed high host lysis activities to control *Samonella* in foods and animals (Hooton et al., 2011; Bardina et al., 2012; Spricigo et al., 2013).

#### Campylobacter jejuni

*C. jejuni* is one of leading causes of zoonotic diseases over the world and about 400–500 million cases of diarrhea are reported each year (Ruiz-Palacios, 2007). Major sources of *C. jejuni* are poultry-originated foods (Wysok and Uradzinski, 2009). To control them, phage treatment has been proposed (Goode et al., 2003; Silva et al., 2011).

Since poultry-originated foods are recognized as a reservoir of *C. jejuni*, most of the phage applications have been focused to reduce bacterial contamination of poultry skin and to inhibit bacterial colonization in poultry intestines. When *C. jejuni* C222 (10^4^ CFU/cm^2^)-contaminated chicken skin was treated with a single *C. jejuni* phage NCTC 12673 (10^6^ PFU/cm^2^), 95% of the contaminated *C. jejuni* was reduced (Goode et al., 2003). In addition, application of *C. jejuni* phage phi2 on the chicken skin showed 2 log reduction. Interestingly, phage phi2 was able to survive on the chicken skin for 10 days, indicating that this phage is very stable and suitable to control *C. jejuni* on poultry (Atterbury et al., 2003).

Treatment of the phage cocktail (CP8 and CP34) revealed 5 log reduction in *C. jejuni* colonization in bird intestines (Loc Carrillo et al., 2005). In addition, a cocktail of three phages (phiCcoIBB35, phiCcoIBB37and phiCcoIBB12) was administered to chicken containing *C. jejuni* and *C. coli* by oral gavage and feeding, resulting in a 2 log reduction in the fecal sample (Carvalho et al., 2010). Interestingly, the cocktail of three phages had a broad lytic spectrum against *Campylobacter*, because three phages showed different and complementary lytic spectra against *C. coli* and *C. jejuni* strains (Carvalho et al., 2010).

#### Listeria monocytogenes

The primary source of *Listeria* are RTE foods, generally preserved in refrigerators, because it can survive and grow in the cold environment. In particular, *L. monocytogenes* is associated with listeriosis outbreaks over the world (Maertens De Noordhout et al., 2014). Although listeriosis is not as common as other food-borne illness, its relatively high fatality rate (about 45%) is a major concern worldwide (Siegman-Igra et al., 2002). Therefore, *L. monocytogenes* should be controlled in foods.

*L. monocytogenes* phage A511 was treated to liquid foods (chocolate milk and mozzarella cheese brine) as well as solid foods (hot dogs, sliced turkey meat, smoked salmon, seafood, sliced cabbage and lettuce leaves). Interestingly, phage A511 treatment to liquid foods reduced the bacterial cells below the detection limit at 6°C for 6 days and the treatment to solid foods also showed 5 log reductions in the same conditions (Guenther et al., 2009). Moreover, A511-like phage FWLLm1 also showed 2.5 log reduction, suggesting that these phages have strong host lysis activity (Bigot et al., 2011). After the FDA approval of phage application in foods (Sharma, 2013), several commercial phage products for food applications were introduced. Listex P100 (Micreos Food Safety) was evaluated to treat cheese during ripening. While this treatment resulted in 3.5 log reduction, high multiplicity of infection (MOI) treatment (>10^8^) was demonstrated to completely eradicate *L. monocytogenes* in cheese (Carlton et al., 2005). In addition, an animal study confirmed that Listex P100 had no toxic effect in animals, suggesting its high safety (Carlton et al., 2005). A cocktail containing two phages, LM103 and LMP-102, exhibited 2–4.6 log reductions in honeydew melon. However, the treatment on apple slices did not show reduction in *L. monocytogenes* and this implies that biocontrol of *L. monocytogenes* using phage cocktails may depend on the kinds of foods despite high host specificity and host lysis activity (Oliveira et al., 2014).

#### Staphylococcus aureus

*S. aureus* is generally found in various foods including sliced meat, salads, pastries, unpasteurized milk, and cheese products. It has been known that this bacterium produces heat stable enterotoxins causing food poisoning such as nausea, vomiting, stomach cramps, and diarrhea (Kadariya et al., 2014). Moreover, emergence of multidrug-resistant *S. aureus* (MRSA) suggests that alternative biocontrol agent need to be developed to replace the use of antibiotics for *S. aureus* treatment (O'Flaherty et al., 2005b; Kadariya et al., 2014).

Interestingly, *S. aureus* phage K is capable of replicating in heat-treated milk but not in raw milk. This is due to heat-labile immunoglobulins preventing adsorption of phage to *S. aureus* in raw milk. Thus, it is suggested that *S. aureus* phages should be applied after heat treatment of milk and milk-associated products (O'Flaherty et al., 2005b). In addition, treatment with a cocktail containing two phages (IPLA35 and IPLA88) on fresh and hard cheeses during curdling process resulted in 3.83 and 4.64 log reductions of *S. aureus*, respectively (Bueno et al., 2012). Despite the cocktail's effectiveness in controlling *S. aureus*, it had no effect on cheese starter strains nor did it alter the chemical properties of cheeses (Bueno et al., 2012). Furthermore, two kinds of phage cocktails (TEAM/P68/LH1-MUT and phi812/44AHJD/phi2) were treated on cheddar cheese curd samples. Interestingly, both phage cocktails completely eradicated a 10^6^ CFU/g of *S. aureus* population at all MOI levels tested (15, 45, and 150) without phage titer reduction. Furthermore, there was no stress-induced enterotoxin C overproduction by *S. aureus* upon phage treatment, implying that phage cocktail application has potential as a *S. aureus*-targeting biocontrol strategy in foods (El Haddad et al., 2016).

#### *Cronobacter sakazakii* and *Vibrio spp*.

*C. sakazakii* is often detected in infant milk powder and is well-known to cause bacteremia, meningitis, and necrotizing enterocolitis. In general, newborn infants are highly susceptible to *C. sakazakii* infection with high fatality rate (Drudy et al., 2006). To evaluate food applications of two different *C. sakazakii* phages, ESP 732-1 and ESP 1-3, were treated to the infant milk formula at three different temperatures (12, 24, and 37°C). Interestingly, phage ESP 732-1 at MOI of 10^7^ eliminated 10^2^ CFU/ml of *C. sakazakii* strain at all tested temperatures, while phage ESP 1-3 inhibited only at 24°C (Kim et al., 2007). This indicates that each *C. sakazakii* phage may have different optimum temperatures. In addition, phage CR5 could completely inhibit both clinical and food *C. sakazakii* isolates with a MOI of 10^5^ (Lee et al., 2016).

*Vibrio* infection is usually associated with eating undercooked seafoods such as oysters (Daniels et al., 2000). Symptoms of vibriosis include watery diarrhea, abdominal cramps, nausea, or fever (Daniels et al., 2000). To control this food-borne pathogen, *Vibrio* phages have been tested in seafood samples. Treatment of *V. parahaemolyticus* phage VPp1 at MOI of 0.1 in the oyster depuration caused 2.35–2.76 log reductions (Cai et al., 2011; Rong et al., 2014). In addition, phage pVp1 at MOI of 10^4^ was effective to control *V. parahaemolyticus* on the surface of oysters with 6 log reductions (Jun et al., 2014a).

### Phage endolysin

Phage endolysin are peptidoglycan hydrolases that play a role in host lysis after phage replication and propagation. Therefore, it has been suggested as a novel biocontrol agent as well as natural food preservative to control food-borne gram-positive pathogens. In this section, general features and various food applications of endolysins will be explained and discussed. Various endolysin applications in foods are summarized in Table 2.

**Table 2:** **Examples of endolysins used in studies related to pathogen reduction in foods**.

**Target host**	**Endolysin**	**Applications**	**Results**	**References**
*Listeria monocytogenes*	Ply500	Iceberg lettuce	Silica nanoparticles (SNPs)-conjugated Ply500 showed 4 log reduction	Solanki et al., 2013
	LysZ5	Soya milk	4 log reduction within 3 h at 4°C	Zhang et al., 2012
*Staphylococcus aureus*	LysH5	Milk	Cell number reduction to under the detected level at 37°C after 4 h	Obeso et al., 2008
	LysH5	Milk	Active in milk when secreted by *Lactococcus lactis*	Rodriguez-Rubio et al., 2012a
	LysH5	Milk	Synergistic inhibition effect combination treatment with nisin	Garcia et al., 2010
	Ply187AN-KSH3b	Milk	Immediate eradication of all CFUs (about 100 CFU) at time zero and remained undetectable throughout the 3 h period	Mao et al., 2013
	λSA2-E-Lyso-SH3b, λSA2-E-LysK-SH3b	Cow milk	Strong activity as lysostaphin reduction in bacterial numbers was maintained during 3 h	Schmelcher et al., 2012b
	HydH5Lyso, HydH5SH3b, CHAPSH3b	Milk	Significantly enhanced lytic activities when compared with parental protein HydH5, CHAPSH3b showed strong lytic activity in both whole and skim milk (pasteurized) under both at 25°C and 37°C	Rodriguez-Rubio et al., 2013
*Clostridium perfrigens*	Ctp1L	Cow milk	Moderate host lysis activity	Mayer et al., 2010; Schrantz et al., 2011

#### General features

After replication and propagation of the *Caudovirales* phages in the host cells, assembled phages are released upon breakdown of bacterial cell wall caused by phage encoded endolysins. Endolysin has a specific activity to hydrolyze peptidoglycan of the cell wall and holin are known to help endolysin to cross the bacterial membrane to reach cell wall (Young and Blasi, 1995). Therefore, endolysin has potential as a biocontrol agent against various Gram-positive food-borne pathogens in the food industry. Generally, endolysin targeting gram-positive bacteria has two conserved protein domains, N-terminal enzymatic activity domain (EAD) and C-terminal cell wall binding domain (CBD). It has been reported that there are five types of EAD according to the cleavage sites: N-aceytlmuramidases (lysozymes), N-acetyl-β-D-glucosaminidases (glycosidases), N-acetylmuramoyl-L-alanine amidases, L-alanoyl-D-glutamate endopeptidases, and interpeptide bridge-specific endopeptidases (Borysowski et al., 2006). Since endolysins specifically target the peptidoglycan layer in the bacteria, they have been considered safe for humans without any immunological responses (Loessner, 2005). Furthermore, no studies on the emergence of endolysin resistance strains has been reported to date (Schmelcher et al., 2012a). Therefore, endolysin may be a good candidate for biocontrol of food-borne pathogens in foods without harming humans. However, most of endolysins are limited to control of gram-positive bacteria and endolysin studies are still at the preliminary stage. To utilize this advantage of endolysins for food applications, further efforts and studies need to be conducted on various food-borne pathogens.

#### Endolysin applications

The endolysin LysZ5 from a *L. monocytogenes* phage FWLLm3 can specifically inhibit the host growth up to 4 log CFU in soya milk within 3 h at 4°C, suggesting that LysZ5 has high host specificity and host lysis activity at refrigerator condition (Zhang et al., 2012). However, listericidal peptidase, Ply500 showed a broad activity spectrum within the genus *Listeria* (Schmelcher et al., 2012c). Interestingly, silica nanoparticles (SNPs)-conjugated Ply500 showed 4 log reduction of *L. innocua* on iceberg lettuce (Solanki et al., 2013). It is noteworthy that this SNPs-conjugated Ply500 revealed significant enzyme stability (retaining >95% of initial host lysis activity) even after 15 days incubation at 25°C, while native endolysin was completely inactivated under the same condition (Solanki et al., 2013). This highlights effectiveness of enzyme immobilization to sustain the activity and stability of the endolysin in food applications.

The endolysin LysH5 from a *S. aureus* phage vB_SauS-phiIPLA88 was demonstrated to inhibit the growth of a broad range of clinical Staphylococcal strains including *S. aureus* and *S. epidermidis* (Obeso et al., 2008). Interestingly, this endolysin could also control biofilm-forming *S. aureus* and *S. epidermidis* strains as well as rifampicin and ciprofloxacin-persister bacteria (Gutierrez et al., 2014). To verify endolysin LysH5's activity in food applications, pasteurized milk containing *S. aureus* was treated with a single endolysin LysH5 or a combination of LysH5 and nisin (Garcia et al., 2010). A synergistic inhibition effect of LysH5 and nisin was observed and this synergistic effect may be associated with these two substances taking different approaches to exert antimicrobial effect (Garcia et al., 2010). Nisin exhibits antimicrobial activity by forming pores in the host membrane, while LysH5 hydrolyzes peptidoglycan. In addition, the phage vB_SauS-phiPLA88 has a highly thermostable HydH5, a peptidoglycan hydrolase domain, to lyse *S. aureus*. Interestingly, HydH5 has two conserved protein domains, N-terminal CHAP domain and a C-terminal LYZ2 domain, but it does not have CBD (Rodriguez-Rubio et al., 2012b). To enhance the host specificity and binding activity, three fusion proteins were constructed: complete HydH5+SH3b domain of lysostaphin (HydH5SH3b), CHAP+SH3b (CHAPSH3b), complete HydH5+complete lysostaphin (HydH5Lyso). Comparative host lysis analysis revealed that the fusion protein containing the CHAP domain of HydH5 and the SH3b domain of lysostaphin showed the strongest host lysis activity in pasteurized milk, suggesting that construction of fused-endolysins through genetic engineering may be necessary to enhance the host lysis activity (Rodriguez-Rubio et al., 2012b). Endolysin LysK from *S. aureus* phage K has a high host lysis activity with broad host spectrum including general and clinical *S. aureus* strains and even MRSA (O'Flaherty et al., 2005a). However, endolysin Ply187 from *S. aureus* phage 187 showed relatively weak host lysis activity, probably due to an inhibitory domain at the C-terminal (Mao et al., 2013). To enhance the host lysis activity of Ply187 without inhibition of host lysis activity, EAD of Ply187 (Ply187AN) was fused to CBD of LysK (KSH3b) to generate a chimeric Ply187AN-KSH3b enzyme. Interestingly, host lysis activity of the fusion protein (Ply187AN-KSH3b) was better than the parental endolysins (Ply187AN and LysK), probably due to removing the inhibitory domain from Ply187 upon fusion. Host lysis test of the contaminated milk samples using the fusion protein (Ply187AN-KSH3b) resulted in no detection of *S. aureus* after 3 h, supporting the advantage of the genetically engineered fusion endolysin (Mao et al., 2013).

The endolysin Ctp1L from *Clostridium* virulent phage phiCTP1 showed host lysis activity against *C. tyrobutyricum* and *C. sporogenes* in cow milk. However, this endolysin showed less host lysis activity in milk sample than in broth condition, indicating effects of food components on endolysin activity should be considered for competitive endolysin application (Mayer et al., 2010; Schrantz et al., 2011).

While many endolysins were screened and characterized, further optimization of their host specificity and host lysis activity is required to maximize the activities. Generation of fused endolysin using genetic engineering may be one of good approaches to achieve this.

## Phage rapid detection of food-borne pathogens in foods

### CBD-based rapid detection in foods

To detect various food-borne pathogens in food samples, three microbiological and molecular methods, such as culturing method using the specific selective media, PCR-based, and antibody-based detection methods, have been generally used. However, these detection methods have some limitations including long incubation time, requirement of expensive molecular techniques, and low sensitivity and stability of antibodies (Wang et al., 2007; Singh et al., 2011). Therefore, development of simple, rapid, and sensitive method for food-borne pathogen detection is required.

As previously explained, endolysin has two conserved protein domains: N-terminal EAD and C-terminal CBD. While EAD is associated with host cell lysis, CBD plays a role in specific host recognition and binding. Due to high host specificity and host-specific binding, CBD can be used to replace antibody for rapid detection of specific bacteria (Rothfuss et al., 2010; Kong et al., 2015). Antibodies have been widely used for rapid detection and concentrating specific food-borne pathogens through specific binding. However, they have high detection limit, their binding specificity for pathogens may sometimes be low (Fratamico et al., 2005), and their production cost is high. Therefore, development of an inexpensive novel material for specific detection and concentration of food-borne pathogens in foods is highly required. The size of CBD (usually 10–20 kDa) is much smaller than that of antibodies (usually 150 kDa) and the number of CBD binding sites on a bacterial cell is reported to be at least 10^7^ suggesting that CBD might be a good candidate to substitute antibodies (Yu et al., 2016). CBD also has advantage of easy construction of fusion proteins containing various fluorescent proteins or other functional domains because it is expressed in bacteria.

Fluorescence-labeled CBD is a good tool to detect specific food-borne pathogen (Loessner et al., 2002). To date, fused CBDs with various fluorescent proteins were developed to target and detect several gram-positive food-borne pathogens including *L. monocytogenes, S. aureus, B. cereus* (Ahmed et al., 2007; Eugster et al., 2011; Kong and Ryu, 2015). However, CBD has a critical limitation as it cannot detect gram-negative food-borne pathogens because of the outer membrane. By using different colored-fluorescent tags, CBD cocktail can identify multiple food-borne pathogens simultaneously present in a food sample. For example, different serovar groups of *Listeria* were identified by a multiplex decoration with different CBDs. Three CBDs, CBD-P35, and CBD500 tagged with different fluorescent markers (RedStar and GFP) were able to distinguish different *Listeria* strains in both milk and camembert cheese samples (Schmelcher et al., 2010).

CBD can also be used for concentration of specific food-borne pathogens in foods. For example, CBD118 and CBD500 from *L. monocytogenes*-targeting endolysins Ply118 and Ply500 were coated on paramagnetic beads and the CBD-coated beads were evaluated for concentration of bacterial cells in various *L. monocytogenes*-contaminated food samples (Kretzer et al., 2007). The concentration of *L. monocytogenes* using these CBD-coated beads showed >90% recovery rate in culture condition. Moreover, these CBD-coated beads captured up to 1–100 CFU/g of *L. monocytogenes* in various food samples including turkey breast, ground meat, salmon, cheese, iceberg lettuce, and milk (Kretzer et al., 2007). Furthermore, CBD from *S. aureus-*targeting endolysin plyV12 was used to concentrate the host cell via immunomagnetic separation method and demonstrated that these CBD-coated beads could detect up to 400 CFU of *S. aureus-*contaminated milk in 1.5 h (Yu et al., 2016). These findings suggest that these bacterial concentration and detection methods of various food-borne pathogens could be implemented for food applications (Yu et al., 2016).

To summarize, CBD could be a great candidate to replace antibodies in rapid detection and concentration of pathogens, because it can overcome limitations of antibody with higher specificity and binding activity for gram-positive pathogens.

### Reporter phage-based rapid detection of live bacteria in foods

Although CBD has high host specificity and binding activity, it is not able to differentiate between live and dead cells. Furthermore, it is impossible to distinguish cell-bound CBD from its free form after CBD treatment without washing step, and it is even very difficult to wash food samples after CBD treatment. To overcome these limitations of CBD, reporter phage has been proposed to detect food-borne pathogens.

Reporter phage is a genetically engineered phage that contains fluorescence-emitting or color-developing gene clusters encoding bacterial luciferase, green fluorescence protein (GFP), and β-galactosidase (Smartt et al., 2012). After recognizing its specific host strain, reporter phage infects the host and injects its genomic DNA (Grayson and Molineux, 2007; Ming et al., 2011). Then, phage DNA is inserted into the host genome and fluorescent or colorimetric signals would be emitted for detection (Schmelcher and Loessner, 2014). The advantage of reporter phage is that it can only emit the detection signal after host infection, indicating that reporter phage detection is restricted to live host cells. Another advantage is that reporter phage emits the detection signal when it infects the specific host, indicating that no washing step is required. Therefore, reporter phage could overcome the limitations of CBD, even though CBD is still a good material for rapid and specific detection of food-borne pathogens in foods.

**Figure 1:**
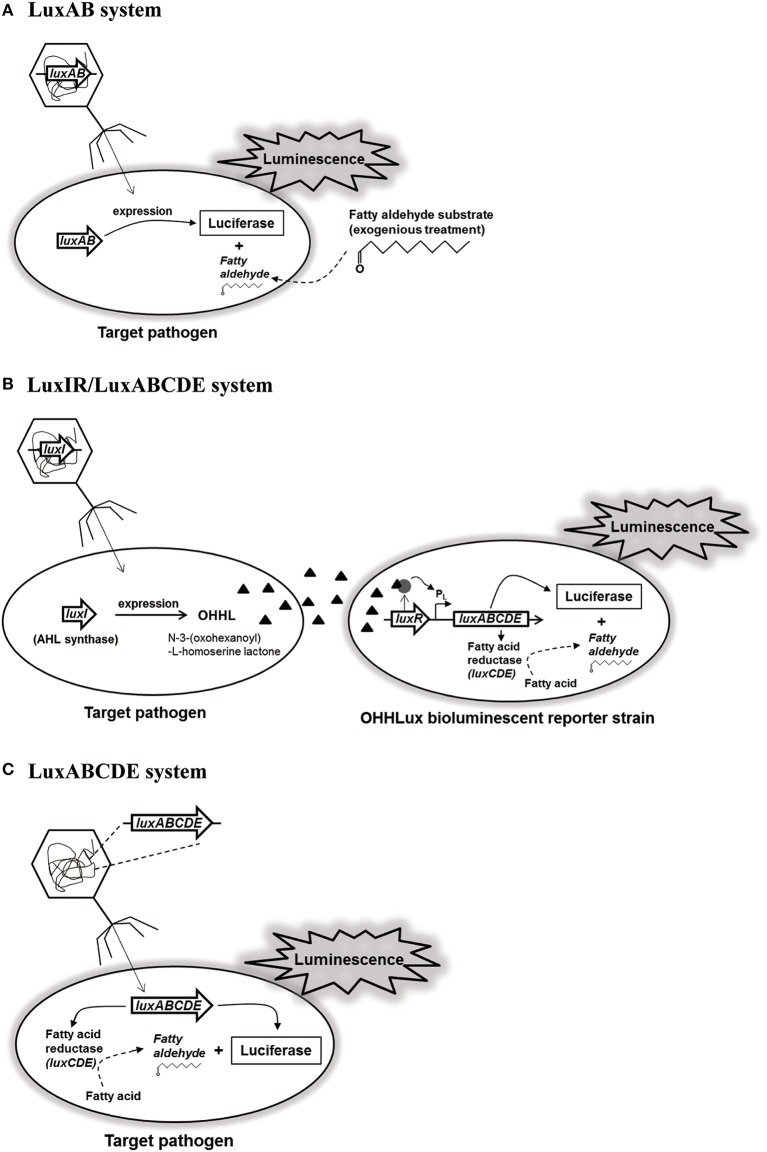
**Luciferase-based reporter phage systems: (A) LuxAB system, (B) LuxIR/LuxABCDE system, and (C) LuxABCDE system**.

Bacterial *lux* gene operon consists of *luxCDE* encoding fatty acid reductase complex containing reductase, synthetase, and transferase for biosynthesis of fatty aldehydes as substrates, and *luxAB* encoding luciferase α- and β-subunits for luminescence reaction with the substrates (Meighen, 1994; Ripp et al., 2008). Based on this *lux* gene operon, a recombinant reporter phage containing only *luxAB* of *V. harveyi* was constructed (Figure 1A). Interestingly, this reporter phage was able to detect six different *L. monocytogenes* strains up to 500–1,000 cells without enrichment step, suggesting high sensitivity and detection ability (Loessner et al., 1996). Although this reporter phage could detect low level of *L. monocytogenes* in various food samples including hamburger, liverwurst, shrimp, pasteurized milk, cheeses, and cabbage, it does not contain *luxCDE* for biosynthesis of fatty aldehydes as substrates for luminescence reaction (Loessner et al., 1997). In addition, *S*. Enteritidis-targeting reporter phage containing only *luxAB* (P22_*luxAB*_) was able to detect up to 63 CFU/egg sample, indicating the requirement of substrate supply for detection (Chen and Griffiths, 1996). Therefore, for easy detection of *E. coli* in food samples without substrate supply, the reporter phage containing *luxI* gene (λ_*luxI*_ phage) and the modified bioluminescent reporter strain containing complete *luxABCDE* operon as well as *luxR* for regulation of operon gene expression (*E. coli* OHHLux) was constructed (Figure 1B). After infection and genome integration of the reporter phage, *luxI* gene was expressed followed by the production of an autoinducer protein (N-3-(oxohexanoyl)-L-homoserine lactone; OHHL). This OHHL can be diffused within bacterial population including the reporter strains. After accepting OHHL, LuxR binds to OHHL and the complex activates the transcription of *luxABCDE* in the operon for luminescence reaction (Ripp et al., 2006). While this detection system can detect up to 1 CFU/ml of *E. coli* in pure culture, it can detect up to 130 CFU/ml in the contaminated lettuce rinsate, suggesting that its sensitivity and detection ability works well even in food samples (Ripp et al., 2006). Furthermore, the other reporter phage/strain detection system (PP01_*luxI*_/*E. coli* OHHLux) targeting *E. coli* O157:H7 could detect up to 1 CFU/ml in pure culture as well as in the food/water samples (apple juice, spinach rinsate, and tap water). However, this system did not work well in the ground beef sample because this sample already had a small amount of OHHL. Therefore, careful selection of food samples for detection is necessary before applying this reporter phage/strain detection system (Ripp et al., 2008). Although this reporter/strain system does not need a supply of substrate, it still needs the reporter strain for detection. To avoid this inconvenience, a new-type *S*. Typhimurium-targeting reporter phage containing a complete set of *luxABCDE* operon (SPC32H-CDABE) was constructed (Kim et al., 2014; Figure 1C). This reporter phage could detect up to 20 CFU/ml of *Salmonella* in pure culture. In addition, its food applications showed that it could detect 22 CFU/g of *Salmonella* in iceberg lettuce, 37 CFU/g of *Salmonella* in sliced pork, and 700 CFU/g of *Salmonella* in milk (Kim et al., 2014). This reporter phage would be useful for monitoring and rapid detection of *S*. Typhimurium in food sample without the supply of substrates or reporter strain for detection.

In addition to the luciferase-based reporter phage systems, a GFP-based reporter phage (PP01-GFP) targeting *E. coli* O157:H7 was constructed (Oda et al., 2004). Although its sensitivity and host range were determined, it was not used for food applications because many food components can be fluorescent. Another reporter phage system was constructed with *lacZ* gene, which encodes β-galactosidase. Interestingly, this *lacZ-*based reporter phage targeting *E. coli* O157:H7 needs specific substrates including chlorophenol red β-D-galactopyranoside (CPRG) for colorimetric reaction and Beta-glo^*TM*^ luminescent substrate (Promega, USA) for luminescence reaction (Willford and Goodridge, 2008). This reporter phage system was utilized for construction of integrated assay with swab for sampling, immunomagnetic beads for separation, *lacZ-*based reporter phage with specific substrates. Its application to beef slice samples showed that its detection limit was up to 10^3^ CFU/100 cm^2^ using colorimetric method and up to 10 CFU/100 cm^2^ using luminescence method, suggesting that it is a good combined rapid detection approach using sampling, separation, and rapid detection (Willford and Goodridge, 2008).

However, it is still necessary to construct various reporter phages because a few food-borne pathogens are detectable using this reporter phage system. Although this reporter phage system can detect only live bacterial cells with high host specificity and low detection limit, construction and development of novel reporter phages are still difficult. Therefore, more study should be needed for development, optimization, and various food application of the reporter phage systems.

## Concluding remarks

During the last century, bacteriophages have been screened and utilized for the purpose of therapy for various diseases caused by pathogens. Since the discovery of antibiotics in Western Europe, the use of phage for therapeutic purposes was reduced and restricted in the area of Eastern Europe. However, emergence of antibiotic-resistant strains, this phage therapy has recently been revisited and reissued. While many phages have been isolated and characterized for application of phage therapy, they also have been considered as novel biocontrol agents to control various food-borne pathogens. For food applications, phages are considered as natural food preservatives as well as rapid detection tools of food-borne pathogens. Numerous studies have been reported showing that phages may be useful for controlling specific food-borne pathogen with high safety for humans. Although phages are very useful and safe for food applications, they are not widely used because they still need some time to make the customers understand the advantage of phages as natural food preservatives. Because of this, well-designed experimental clinical studies should be performed to convince the customers that phage is highly safe and no harm to humans. To overcome the low preference to phage applications, endolysin with high host specificity and lysis activity has been suggested and developed. However, this enzyme still needs to be optimized experimentally and enhanced by molecular protein engineering. For rapid detection of food-borne pathogens in foods, PCR- and antibody-based methods are generally used. However, these detection methods still have some problems including long reaction time, no knowledge of molecular techniques, high detection limit, etc. Based on the host specificity of phages, CBD from endolysin and reporter phage system have been suggested to detect food-borne pathogens for rapid detection without additional equipment or reagents, suggesting the next-generation rapid detection system of pathogens. Furthermore, CBD has similar characteristics including high specificity and binding activity to antibodies. Therefore, CBD has recently been suggested to be an alternative material of antibodies because CBD is much cheaper to be produced using *E. coli* overexpression/ purification system with much higher host specificity and binding activity than antibody. Therefore, antibody in some parts of the market may sooner or later be replaced by CBD. Although reporter phage construction is not simple at this time, one it is obtained, its detection is quite quick with low detection limit and very simple. In addition, reporter phage can detect only live pathogens. Therefore, reporter phage detection system may be suitable for development of commercial rapid detection kit in the future.

## Author contributions

Conceived and designed the review contents: SR, JL. Literature reading and review: JB, YK, SR, JL. Wrote the paper: JB, YK, SR, and JL.

### Conflict of interest statement

The authors declare that the research was conducted in the absence of any commercial or financial relationships that could be construed as a potential conflict of interest.

## References

[B1] Abuladze T. Li M. Menetrez M. Y. Dean T. Senecal A. Sulakvelidze A. (2008). Bacteriophages reduce experimental contamination of hard surfaces, tomato, spinach, broccoli, and ground beef by *Escherichia coli* O157:H7. Appl. Environ. Microbiol. 74, 6230–6238. 10.1128/AEM.01465-08 18723643 PMC2570303

[B2] Ackermann H. W. (1998). Tailed bacteriophages: the order caudovirales. Adv. Virus Res. 51, 135–201. 10.1016/S0065-3527(08)60785-X 9891587 PMC7173057

[B3] Ahmed A. B. F. Noguchi K. Asami Y. Nomura K. Fujii H. Sakata M.. (2007). Evaluation of cell wall binding domain of *Staphylococcus aureus* autolysin as affinity reagent for bacteria and its application to bacterial detection. J. Biosci. Bioeng. 104, 55–61. 10.1263/jbb.104.55 17697984

[B4] Atterbury R. J. Connerton P. L. Dodd C. E. Rees C. E. Connerton I. F. (2003). Application of host-specific bacteriophages to the surface of chicken skin leads to a reduction in recovery of *Campylobacter jejuni*. Appl. Environ. Microbiol. 69, 6302–6306. 10.1128/AEM.69.10.6302-6306.2003 14532096 PMC201188

[B5] Bao H. D. Zhang P. Y. Zhang H. Zhou Y. Zhang L. L. Wang R. (2015). Bio-control of *Salmonella* Enteritidis in foods using bacteriophages. Viruses 7, 4836–4853. 10.3390/v7082847 26305252 PMC4576208

[B6] Bardina C. Spricigo D. A. Cortes P. Llagostera M. (2012). Significance of the bacteriophage treatment schedule in reducing *Salmonella* colonization of poultry. Appl. Environ. Microbiol. 78, 6600–6607. 10.1128/AEM.01257-12 22773654 PMC3426709

[B7] Bertin A. De Frutos M. Letellier L. (2011). Bacteriophage-host interactions leading to genome internalization. Curr. Opin. Microbiol. 14, 492–496. 10.1016/j.mib.2011.07.010 21783404

[B8] Bigot B. Lee W. J. McIntyre L. Wilson T. Hudson J. A. Billington C.. (2011). Control of *Listeria monocytogenes* growth in a ready-to-eat poultry product using a bacteriophage. Food Microbiol. 28, 1448–1452. 10.1016/j.fm.2011.07.001 21925027

[B9] Borysowski J. Weber-Dabrowska B. Gorski A. (2006). Bacteriophage endolysins as a novel class of antibacterial agents. Exp. Biol. Med. 231, 366–377. 16565432 10.1177/153537020623100402

[B10] Bueno E. Garcia P. Martinez B. Rodriguez A. (2012). Phage inactivation of *Staphylococcus aureus* in fresh and hard-type cheeses. Int. J. Food Microbiol. 158, 23–27. 10.1016/j.ijfoodmicro.2012.06.012 22795798

[B11] Cai L. Zhan R. Pu K. Y. Qi X. Zhang H. Huang W.. (2011). Butterfly-shaped conjugated oligoelectrolyte/graphene oxide integrated assay for light-up visual detection of heparin. Anal. Chem. 83, 7849–7855. 10.1021/ac2016135 21882808

[B12] Carlton R. M. Noordman W. H. Biswas B. de Meester E. D. Loessner M. J. (2005). Bacteriophage P100 for control of *Listeria monocytogenes* in foods: genome sequence, bioinformatic analyses, oral toxicity study, and application. Regul. Toxicol. Pharmacol. 43, 301–312. 10.1016/j.yrtph.2005.08.005 16188359

[B13] Carvalho C. M. Gannon B. W. Halfhide D. E. Santos S. B. Hayes C. M. Roe J. M.. (2010). The *in vivo* efficacy of two administration routes of a phage cocktail to reduce numbers of *Campylobacter coli* and *Campylobacter jejuni* in chickens. BMC Microbiol. 10:232. 10.1186/1471-2180-10-232 20809975 PMC2940857

[B14] Chang Y. Shin H. Lee J. H. Park C. J. Paik S. Y. Ryu S. (2015). Isolation and genome characterization of the virulent *Staphylococcus aureus* bacteriophage SA97. Viruses 7, 5225–5242. 10.3390/v7102870 26437428 PMC4632378

[B15] Chen J. Griffiths M. W. (1996). *Salmonella* detection in eggs using Lux(+) bacteriophages. J. Food Prot. 59, 908–914. 10.4315/0362-028X-59.9.908 31159106

[B16] Clark J. R. March J. B. (2006). Bacteriophages and biotechnology: vaccines, gene therapy and antibacterials. Trends Biotechnol. 24, 212–218. 10.1016/j.tibtech.2006.03.003 16567009

[B17] Cody S. H. Glynn M. K. Farrar J. A. Cairns K. L. Griffin P. M. Kobayashi J.. (1999). An outbreak of *Escherichia coli* O157 : H7 infection from unpasteurized commercial apple juice. Ann. Internl. Med. 130, 202–209. 10.7326/0003-4819-130-3-199902020-00005 10049198

[B18] Coffey B. Rivas L. Duffy G. Coffey A. Ross R. P. McAuliffe O. (2011). Assessment of *Escherichia coli* O157:H7-specific bacteriophages e11/2 and e4/1c in model broth and hide environments. Int. J. Food Microbiol. 147, 188–194. 10.1016/j.ijfoodmicro.2011.04.001 21531032

[B19] Daniels N. A. MacKinnon L. Bishop R. Altekruse S. Ray B. Hammond R. M.. (2000). *Vibrio parahaemolyticus* infections in the United States, 1973-1998. J. Infect. Dis. 181, 1661–1666. 10.1086/315459 10823766

[B20] Dennis A. W. Siu V. M. Campagnolo C. Soldin S. J. (2010). Interference in an unconjugated estriol assay causing a false negative integrated prenatal screening report. Prenat. Diagn. 30, 165–167. 10.1002/pd.2426 20020420

[B21] Deresinski S. (2009). Bacteriophage therapy: exploiting smaller fleas. Clin. Infect. Dis. 48, 1096–1101. 10.1086/597405 19275495

[B22] Devreese K. M. (2010). No more mixing tests required for integrated assay systems in the laboratory diagnosis of lupus anticoagulants? J. Thromb. Haemost. 8, 1120–1122. 10.1111/j.1538-7836.2010.03807.x 20149080

[B23] d'Hérelle F. (1917). Sur un microbe invisible antagoniste des bacilles dysentérique. Acad. Sci. Paris. 165, 373–375.

[B24] Drudy D. Mullane N. R. Quinn T. Wall P. G. Fanning S. (2006). *Enterobacter sakazakii*: an emerging pathogen in powdered infant formula. Clin. Infect. Dis. 42, 996–1002. 10.1086/501019 16511766

[B25] Duckworth D. H. (1976). Who discovered bacteriophage? Bacteriol. Rev. 40, 793–802. 795414 10.1128/br.40.4.793-802.1976 PMC413985

[B26] El-Shibiny A. Scott A. Timms A. Metawea Y. Connerton P. Connerton I. (2009). Application of a group II *Campylobacter* bacteriophage to reduce strains of *Campylobacter jejuni* and *Campylobacter coli* colonizing broiler chickens. J. Food Prot. 72, 733–740. 19435220 10.4315/0362-028x-72.4.733

[B27] El Haddad L. Roy J. P. Khalil G. E. St-Gelais D. Champagne C. P. Labrie S.. (2016). Efficacy of two *Staphylococcus aureus* phage cocktails in cheese production. Int. J. Food Microbiol. 217, 7–13. 10.1016/j.ijfoodmicro.2015.10.001 26476571

[B28] Endersen L. O'Mahony J. Hill C. Ross R. P. McAuliffe O. Coffey A. (2014). Phage therapy in the food industry. Annu. Rev. Food Sci. Technol. 5, 327–349. 10.1146/annurev-food-030713-092415 24422588

[B29] Eugster M. R. Haug M. C. Huwiler S. G. Loessner M. J. (2011). The cell wall binding domain of *Listeria* bacteriophage endolysin PlyP35 recognizes terminal GlcNAc residues in cell wall teichoic acid. Mol. Microbiol. 81, 1419–1432. 10.1111/j.1365-2958.2011.07774.x 21790805

[B30] Fratamico P. Bayles D. Bhunia A. Smith J. (2005). Molecular Approaches for Detection, Identification, and Analysis of Foodborne Pathogens. Boca Raton; CRC Press LLC.

[B31] Garcia P. Martinez B. Rodriguez L. Rodriguez A. (2010). Synergy between the phage endolysin LysH5 and nisin to kill *Staphylococcus aureus* in pasteurized milk. Int. J. Food Microbiol. 141, 151–155. 10.1016/j.ijfoodmicro.2010.04.029 20537744

[B32] Giusiano S. Formisano-Treziny C. Benziane A. Maroc N. Picard C. Hermitte F.. (2010). Development of a biochip-based assay integrated in a global strategy for identification of fusion transcripts in acute myeloid leukemia: a work flow for acute myeloid leukemia diagnosis. Int. J. Lab. Hematol. 32, 398–409. 10.1111/j.1751-553X.2009.01201.x 19930410

[B33] Goode D. Allen V. M. Barrow P. A. (2003). Reduction of experimental *Salmonella* and *Campylobacter* contamination of chicken skin by application of lytic bacteriophages. Appl. Environ. Microbiol. 69, 5032–5036. 10.1128/AEM.69.8.5032-5036.2003 12902308 PMC169133

[B34] Grayson P. Molineux I. J. (2007). Is phage DNA 'injected' into cells-biologists and physicists can agree. Curr. Opin. Microbiol. 10, 401–409. 10.1016/j.mib.2007.04.004 17714979 PMC2064038

[B35] Guenther S. Herzig O. Fieseler L. Klumpp J. Loessner M. J. (2012). Biocontrol of *Salmonella* Typhimurium in RTE foods with the virulent bacteriophage FO1-E2. Int. J. Food Microbiol. 154, 66–72. 10.1016/j.ijfoodmicro.2011.12.023 22244192

[B36] Guenther S. Huwyler D. Richard S. Loessner M. J. (2009). Virulent bacteriophage for efficient biocontrol of *Listeria monocytogenes* in Ready-To-Eat Foods. Appl. Environ. Microbiol. 75, 93–100. 10.1128/AEM.01711-08 19011076 PMC2612219

[B37] Guenther S. Loessner M. J. (2011). Bacteriophage biocontrol of *Listeria monocytogenes* on soft ripened white mold and red-smear cheeses. Bacteriophage 1, 94–100. 10.4161/bact.1.2.15662 22334865 PMC3278646

[B38] Gutierrez D. Ruas-Madiedo P. Martinez B. Rodriguez A. Garcia P. (2014). Effective removal of *Staphylococcal* biofilms by the endolysin LysH5. PLoS ONE 9:e107307. 10.1371/journal.pone.0107307 25203125 PMC4159335

[B39] Hagens S. Loessner M. J. (2007). Application of bacteriophages for detection and control of foodborne pathogens. Appl. Microbiol. Biotechnol. 76, 513–519. 10.1007/s00253-007-1031-8 17554535

[B40] Hertwig S. Hammerl J. A. Appel B. Alter T. (2013). Post-harvest application of lytic bacteriophages for biocontrol of foodborne pathogens and spoilage bacteria. Berl. Munch. Tierarztl. Wochenschr. 126, 357–369. 24199377

[B41] Hooton S. P. Atterbury R. J. Connerton I. F. (2011). Application of a bacteriophage cocktail to reduce *Salmonella* Typhimurium U288 contamination on pig skin. Int. J. Food Microbiol. 151, 157–163. 10.1016/j.ijfoodmicro.2011.08.015 21899907

[B42] Hudson J. A. Billington C. Cornelius A. J. Wilson T. On S. L. W. Premaratne A.. (2013). Use of a bacteriophage to inactivate *Escherichia coli* O157:H7 on beef. Food Microbiol. 36, 14–21. 10.1016/j.fm.2013.03.006 23764215

[B43] Inal J. M. (2003). Phage therapy: a reappraisal of bacteriophages as antibiotics. Arch. Immunol. Ther. Exp. 51, 237–244. 12956433

[B44] Jado I. Lopez R. Garcia E. Fenoll A. Casal J. Garcia P.. (2003). Phage lytic enzymes as therapy for antibiotic-resistant *Streptococcus pneumoniae* infection in a murine sepsis model. J. Antimicrob. Chemother. 52, 967–973. 10.1093/jac/dkg485 14613958

[B45] Jun J. W. Kim H. J. Yun S. K. Chai J. Y. Park S. C. (2014a). Eating oysters without risk of vibriosis: application of a bacteriophage against *Vibrio parahaemolyticus* in oysters. Int. J. Food. Microbiol. 188, 31–35. 10.1016/j.ijfoodmicro.2014.07.007 25086350

[B46] Jun S. Y. Jung G. M. Yoon S. J. Choi Y. J. Koh W. S. Moon K. S.. (2014b). Preclinical safety evaluation of intravenously administered SAL200 containing the recombinant phage endolysin SAL-1 as a pharmaceutical ingredient. Antimicrob. Agents Chemother. 58, 2084–2088. 10.1128/AAC.02232-13 24449776 PMC4023757

[B47] Juneja V. K. Dwivedi H. P. Yan X. (2012). Novel natural food antimicrobials. Annu. Rev. Food Sci. Technol. 3, 381–403. 10.1146/annurev-food-022811-101241 22385168

[B48] Kadariya J. Smith T. C. Thapaliya D. (2014). *Staphylococcus aureus* and staphylococcal food-borne disease: an ongoing challenge in public health. BioMed Res. Int. 2014:827965. 10.1155/2014/827965 24804250 PMC3988705

[B49] Kang H. W. Kim J. W. Jung T. S. Woo G. J. (2013). wksl3, a New biocontrol agent for *Salmonella enterica* serovars Enteritidis and Typhimurium in foods: characterization, application, sequence analysis, and oral acute toxicity study. Appl. Environ. Microbiol. 79, 1956–1968. 10.1128/AEM.02793-12 23335772 PMC3592225

[B50] Kaper J. B. (1998). Enterohemorrhagic *Escherichia coli*. Curr. Opin. Microbiol. 1, 103–108. 10.1016/S1369-5274(98)80149-5 10066458

[B51] Kim K. P. Klumpp J. Loessner M. J. (2007). *Enterobacter sakazakii* bacteriophages can prevent bacterial growth in reconstituted infant formula. Int. J. Food Microbiol. 115, 195–203. 10.1016/j.ijfoodmicro.2006.10.029 17196280

[B52] Kim S. Kim M. Ryu S. (2014). Development of an engineered bioluminescent reporter phage for the sensitive detection of viable *Salmonella* Typhimurium. Anal. Chem. 86, 5858–5864. 10.1021/ac500645c 24806327

[B53] Kong M. Ryu S. (2015). Bacteriophage PBC1 and its endolysin as an antimicrobial agent against *Bacillus cereus*. Appl. Environ. Microbiol. 81, 2274–2283. 10.1128/AEM.03485-14 25595773 PMC4357929

[B54] Kong M. Sim J. Kang T. Nguyen H. H. Park H. K. Chung B. H.. (2015). A novel and highly specific phage endolysin cell wall binding domain for detection of *Bacillus cereus*. Eur. Biophys. J. 44, 437–446. 10.1007/s00249-015-1044-7 26043681

[B55] Kretzer J. W. Lehmann R. Schmelcher M. Banz M. Kim K. P. Korn C.. (2007). Use of high-affinity cell wall-binding domains of bacteriophage endolysins for immobilization and separation of bacterial cells. Appl. Environ. Microbiol. 73, 1992–2000. 10.1128/AEM.02402-06 17277212 PMC1828835

[B56] Kutter E. Sulakvelidze A. (2005). Basic phage biology, in Bacteriophages : Biology and Applications, ed Kutter E. (Boca Raton, FL: CRC Press), 375–405.

[B57] Lee J. H. Bai J. Shin H. Kim Y. Park B. Heu S.. (2016). A novel bacteriophage targeting *Cronobacter sakazakii* is a potential biocontrol agent in foods. Appl. Environ. Microbiol. 82, 192–201. 10.1128/AEM.01827-15 26497465 PMC4702651

[B58] Leverentz B. Conway W. S. Janisiewicz W. Camp M. J. (2004). Optimizing concentration and timing of a phage spray application to reduce *Listeria monocytogenes* on honeydew melon tissue. J. Food Prot. 67, 1682–1686. 15330534 10.4315/0362-028x-67.8.1682

[B59] Li D. Gu A. Z. Yang W. He M. Hu X. H. Shi H. C. (2010). An integrated cell culture and reverse transcription quantitative PCR assay for detection of infectious rotaviruses in environmental waters. J. Microbiol. Methods 82, 59–63. 10.1016/j.mimet.2010.04.003 20399813

[B60] Loc Carrillo C. Atterbury R. J. el-Shibiny A. Connerton P. L. Dillon E. Scott A.. (2005). Bacteriophage therapy to reduce *Campylobacter jejuni* colonization of broiler chickens. Appl. Environ. Microbiol. 71, 6554–6563. 10.1128/AEM.71.11.6554-6563.2005 16269681 PMC1287621

[B61] Loessner M. J. (2005). Bacteriophage endolysins—current state of research and applications. Curr. Opin. Microbiol. 8, 480–487. 10.1016/j.mib.2005.06.002 15979390

[B62] Loessner M. J. Kramer K. Ebel F. Scherer S. (2002). C-terminal domains of *Listeria monocytogenes* bacteriophage murein hydrolases determine specific recognition and high-affinity binding to bacterial cell wall carbohydrates. Mol. Microbiol. 44, 335–349. 10.1046/j.1365-2958.2002.02889.x 11972774

[B63] Loessner M. J. Rees C. E. Stewart G. S. Scherer S. (1996). Construction of luciferase reporter bacteriophage A511::luxAB for rapid and sensitive detection of viable *Listeria* cells. Appl. Environ. Microbiol. 62, 1133–1140. 8919773 10.1128/aem.62.4.1133-1140.1996 PMC167878

[B64] Loessner M. J. Rudolf M. Scherer S. (1997). Evaluation of luciferase reporter bacteriophage A511::luxAB for detection of *Listeria monocytogenes* in contaminated foods. Appl. Environ. Microbiol. 63, 2961–2965. 9251182 10.1128/aem.63.8.2961-2965.1997 PMC168593

[B65] Ma B. Zhang G. Qin J. Lin B. (2009). Characterization of drug metabolites and cytotoxicity assay simultaneously using an integrated microfluidic device. Lab Chip 9, 232–238. 10.1039/B809117J 19107278

[B66] Maertens De Noordhout C. Devleesschauwer B. Angulo F. J. Verbeke G. Haagsma J. Kirk M.. (2014). The global burden of listeriosis: a systematic review and meta-analysis. Lancet Infect. Dis. 14, 1073–1082. 10.1016/S1473-3099(14)70870-9 25241232 PMC4369580

[B67] Mao J. Z. Schmelcher M. Harty W. J. Foster-Frey J. Donovan D. M. (2013). Chimeric Ply187 endolysin kills *Staphylococcus aureus* more effectively than the parental enzyme. FEMS Microbiol. Lett. 342, 30–36. 10.1111/1574-6968.12104 23413880 PMC3690576

[B68] Martínez-Díaz S. F. Hipólito-Morales A. (2013). Efficacy of phage therapy to prevent mortality during the vibriosis of brine shrimp. Aquaculture 400–401, 120–124. 10.1016/j.aquaculture.2013.03.007

[B69] Mayer M. J. Payne J. Gasson M. J. Narbad A. (2010). Genomic sequence and characterization of the virulent bacteriophage phiCTP1 from Clostridium tyrobutyricum and heterologous expression of its endolysin. Appl. Environ. Microbiol. 76, 5415–5422. 10.1128/AEM.00989-10 20581196 PMC2918958

[B70] McCallin S. Alam Sarker S. Barretto C. Sultana S. Berger B. Huq S.. (2013). Safety analysis of a Russian phage cocktail: from metagenomic analysis to oral application in healthy human subjects. Virology 443, 187–196. 10.1016/j.virol.2013.05.022 23755967

[B71] Meighen E. A. (1994). Genetics of bacterial bioluminescence. Annu. Rev. Genet. 28, 117–139. 10.1146/annurev.ge.28.120194.001001 7893120

[B72] Ming H. X. Zhu L. Zhang Y. (2011). Rapid quantification of infectious enterovirus from surface water in Bohai Bay, China using an integrated cell culture-qPCR assay. Mar. Pollut. Bull. 62, 2047–2054. 10.1016/j.marpolbul.2011.07.024 21889173

[B73] Nelson D. C. Schmelcher M. Rodriguez-Rubio L. Klumpp J. Pritchard D. G. Dong S. L.. (2012). Endolysins as Antimicrobials. Adv. Virus Res. 83, 299–365. 10.1016/B978-0-12-394438-2.00007-4 22748813

[B74] Newell D. G. Koopmans M. Verhoef L. Duizer E. Aidara-Kane A. Sprong H.. (2010). Food-borne diseases - the challenges of 20 years ago still persist while new ones continue to emerge. Int. J. Food Microbiol. 139 (Suppl. 1), S3–S15. 10.1016/j.ijfoodmicro.2010.01.021 20153070 PMC7132498

[B75] O'Flaherty S. Coffey A. Meaney W. Fitzgerald G. F. Ross R. P. (2005a). The recombinant phage lysin LysK has a broad spectrum of lytic activity against clinically relevant Staphylococci, including methicillin-resistant *Staphylococcus aureus*. J. Bacteriol. 187, 7161–7164. 10.1128/JB.187.20.7161-7164.2005 16199588 PMC1251611

[B76] O'Flaherty S. Coffey A. Meaney W. J. Fitzgerald G. F. Ross R. P. (2005b). Inhibition of bacteriophage K proliferation on *Staphylococcus aureus* in raw bovine milk. Lett. Appl. Microbiol. 41, 274–279. 10.1111/j.1472-765X.2005.01762.x 16108920

[B77] O'flaherty S. Ross R. P. Meaney W. Fitzgerald G. F. Elbreki M. F. Coffey A. (2005c). Potential of the polyvalent anti-Staphylococcus bacteriophage K for control of antibiotic-resistant staphylococci from hospitals. Appl. Environ. Microbiol. 71, 1836–1842. 10.1128/AEM.71.4.1836-1842.2005 15812009 PMC1082512

[B78] O'Flynn G. Ross R. P. Fitzgerald G. F. Coffey A. (2004). Evaluation of a cocktail of three bacteriophages for biocontrol of *Escherichia coli* O157:H7. Appl. Environ. Microbiol. 70, 3417–3424. 10.1128/AEM.70.6.3417-3424.2004 15184139 PMC427753

[B79] Obeso J. M. Martinez B. Rodriguez A. Garcia P. (2008). Lytic activity of the recombinant staphylococcal bacteriophage PhiH5 endolysin active against *Staphylococcus aureus* in milk. Int. J. Food Microbiol. 128, 212–218. 10.1016/j.ijfoodmicro.2008.08.010 18809219

[B80] Oda M. Morita M. Unno H. Tanji Y. (2004). Rapid detection of *Escherichia coli* O157:H7 by using green fluorescent protein-labeled PP01 bacteriophage. Appl. Environ. Microbiol. 70, 527–534. 10.1128/AEM.70.1.527-534.2004 14711684 PMC321238

[B81] Oliveira M. Vinas I. Colas P. Anguera M. Usall J. Abadias M. (2014). Effectiveness of a bacteriophage in reducing *Listeria monocytogenes* on fresh-cut fruits and fruit juices. Food Microbiol. 38, 137–142. 10.1016/j.fm.2013.08.018 24290636

[B82] Pawlowska A. M. Zannini E. Coffey A. Arendt E. K. (2012). “Green preservatives”: combating fungi in the food and feed industry by applying antifungal lactic acid bacteria. Adv. Food Nutr. Res. 66, 217–238. 10.1016/B978-0-12-394597-6.00005-7 22909981

[B83] Plym Forshell L. Wierup M. (2006). *Salmonella* contamination: a significant challenge to the global marketing of animal food products. Rev. Sci. Tech. 25, 541–554. 10.20506/rst.25.2.1683 17094696

[B84] Ripp S. Jegier P. Birmele M. Johnson C. M. Daumer K. A. Garland J. L.. (2006). Linking bacteriophage infection to quorum sensing signalling and bioluminescent bioreporter monitoring for direct detection of bacterial agents. J. Appl. Microbiol. 100, 488–499. 10.1111/j.1365-2672.2005.02828.x 16478488

[B85] Ripp S. Jegier P. Johnson C. M. Brigati J. R. Sayler G. S. (2008). Bacteriophage-amplified bioluminescent sensing of *Escherichia coli* O157:H7. Anal. Bioanal. Chem. 391, 507–514. 10.1007/s00216-007-1812-z 18188543

[B86] Rivas L. Coffey B. McAuliffe O. McDonnell M. J. Burgess C. M. Coffey A.. (2010). *In vivo* and *ex vivo* evaluations of bacteriophages e11/2 and e4/1c for use in the control of *Escherichia coli* O157:H7. Appl. Environ. Microbiol. 76, 7210–7216. 10.1128/AEM.01530-10 20851992 PMC2976219

[B87] Rodriguez-Rubio L. Gutierrez D. Martinez B. Rodriguez A. Garcia P. (2012a). Lytic activity of LysH5 endolysin secreted by *Lactococcus lactis* using the secretion signal sequence of bacteriocin Lcn972. Appl. Environ. Microbiol. 78, 3469–3472. 10.1128/AEM.00018-12 22344638 PMC3346474

[B88] Rodriguez-Rubio L. Martinez B. Donovan D. M. Garcia P. Rodriguez A. (2013). Potential of the virion-associated peptidoglycan hydrolase HydH5 and its derivative fusion proteins in milk biopreservation. PLoS ONE 8:e54828. 10.1371/journal.pone.0054828 23359813 PMC3554637

[B89] Rodriguez-Rubio L. Martinez B. Rodriguez A. Donovan D. M. Garcia P. (2012b). Enhanced staphylolytic activity of the *Staphylococcus aureus* bacteriophage vB_SauS-phiIPLA88 HydH5 virion-associated peptidoglycan hydrolase: fusions, deletions, and synergy with LysH5. Appl. Environ. Microbiol. 78, 2241–2248. 10.1128/AEM.07621-11 22267667 PMC3302612

[B90] Rong R. Lin H. Wang J. X. Khan M. N. Li M. (2014). Reductions of Vibrio parahaemolyticus in oysters after bacteriophage application during depuration. Aquaculture 418, 171–176. 10.1016/j.aquaculture.2013.09.028

[B91] Rothfuss A. O'donovan M. De Boeck M. Brault D. Czich A. Custer L.. (2010). Collaborative study on fifteen compounds in the rat-liver Comet assay integrated into 2- and 4-week repeat-dose studies. Mutat. Res. 702, 40–69. 10.1016/j.mrgentox.2010.07.006 20656055

[B92] Ruiz-Palacios G. M. (2007). The health burden of *Campylobacter* infection and the impact of antimicrobial resistance: playing chicken. Clin. Infect. Dis. 44, 701–703. 10.1086/509936 17278063

[B93] Sarhan W. A. Azzazy H. M. E. (2015). Phage approved in food, why not as a therapeutic? Expert Rev. Anti Infect. Ther. 13, 91–101. 10.1586/14787210.2015.990383 25488141

[B94] Scallan E. Hoekstra R. M. Angulo F. J. Tauxe R. V. Widdowson M. A. Roy S. L.. (2011). Foodborne illness acquired in the United States-major pathogens. Emerg. Infect. Dis. 17, 7–15. 10.3201/eid1701.P11101 21192848 PMC3375761

[B95] Schmelcher M. Donovan D. M. Loessner M. J. (2012a). Bacteriophage endolysins as novel antimicrobials. Future Microbiol. 7, 1147–1171. 10.2217/fmb.12.97 23030422 PMC3563964

[B96] Schmelcher M. Loessner M. J. (2014). Application of bacteriophages for detection of foodborne pathogens. Bacteriophage 4, e28137. 10.4161/bact.28137 24533229 PMC3919822

[B97] Schmelcher M. Loessner M. J. (2015). Bacteriophage endolysins: applications for food safety. Curr. Opin. Biotechnol. 37, 76–87. 10.1016/j.copbio.2015.10.005 26707470

[B98] Schmelcher M. Powell A. M. Becker S. C. Camp M. J. Donovan D. M. (2012b). Chimeric phage lysins act synergistically with lysostaphin to kill mastitis-causing *Staphylococcus aureus* in murine mammary glands. Appl. Environ. Microbiol. 78, 2297–2305. 10.1128/AEM.07050-11 22286996 PMC3302589

[B99] Schmelcher M. Shabarova T. Eugster M. R. Eichenseher F. Tchang V. S. Banz M.. (2010). Rapid multiplex detection and differentiation of *Listeria* Cells by use of fluorescent phage endolysin cell wall binding domains. Appl. Environ. Microbiol. 76, 5745–5756. 10.1128/AEM.00801-10 20622130 PMC2935047

[B100] Schmelcher M. Waldherr F. Loessner M. J. (2012c). *Listeria* bacteriophage peptidoglycan hydrolases feature high thermoresistance and reveal increased activity after divalent metal cation substitution. Appl. Microbiol. Biotechnol. 93, 633–643. 10.1007/s00253-011-3372-6 21720825

[B101] Schrantz S. J. Babcock C. A. Theodosis C. Brown S. Mercer S. Pillow M. T.. (2011). A targeted, conventional assay, emergency department HIV testing program integrated with existing clinical procedures. Ann. Emerg. Med. 58, S85–88e81. 10.1016/j.annemergmed.2011.03.031 21684415

[B102] Sharma M. (2013). Lytic bacteriophages: potential interventions against enteric bacterial pathogens on produce. Bacteriophage 3, e25518. 10.4161/bact.25518 24228223 PMC3821672

[B103] Siegman-Igra Y. Levin R. Weinberger M. Golan Y. Schwartz D. Samra Z.. (2002). *Listeria monocytogenes* infection in Israel and review of cases worldwide. Emerg. Infect. Dis. 8, 305–310. 10.3201/eid0803.010195 11927029 PMC3369577

[B104] Silva J. Leite D. Fernandes M. Mena C. Gibbs P. A. Teixeira P. (2011). Campylobacter spp. as a foodborne pathogen: a review. Front. Microbiol. 2:200. 10.3389/fmicb.2011.00200 21991264 PMC3180643

[B105] Singh A. Arutyunov D. McDermott M. T. Szymanski C. M. Evoy S. (2011). Specific detection of *Campylobacter jejuni* using the bacteriophage NCTC 12673 receptor binding protein as a probe. Analyst 136, 4780–4786. 10.1039/c1an15547d 21955997

[B106] Smartt A. E. Xu T. T. Jegier P. Carswell J. J. Blount S. A. Sayler G. S.. (2012). Pathogen detection using engineered bacteriophages. Anal. Bioanal. Chem. 402, 3127–3146. 10.1007/s00216-011-5555-5 22101465

[B107] Solanki K. Grover N. Downs P. Paskaleva E. E. Mehta K. K. Lee L.. (2013). Enzyme-based listericidal nanocomposites. Sci. Rep. 3, 1584. 10.1038/srep01584 23545700 PMC3613805

[B108] Spricigo D. A. Bardina C. Cortes P. Llagostera M. (2013). Use of a bacteriophage cocktail to control *Salmonella* in food and the food industry. Int. J. Food Microbiol. 165, 169–174. 10.1016/j.ijfoodmicro.2013.05.009 23735218

[B109] Sulakvelidze A. (2013). Using lytic bacteriophages to eliminate or significantly reduce contamination of food by foodborne bacterial pathogens. J. Sci. Food Agric. 93, 3137–3146. 10.1002/jsfa.6222 23670852

[B110] Tomat D. Mercanti D. Balague C. Quiberoni A. (2013a). Phage biocontrol of enteropathogenic and *Shiga* toxin-producing *Escherichia coli* during milk fermentation. Lett. Appl. Microbiol. 57, 3–10. 10.1111/lam.12074 23551112

[B111] Tomat D. Migliore L. Aquili V. Quiberoni A. Balague C. (2013b). Phage biocontrol of enteropathogenic and shiga toxin-producing *Escherichia coli* in meat products. Front. Cell. Infect. Microbiol. 3:10. 10.3389/fcimb.2013.00020 23761050 PMC3674477

[B112] Viazis S. Akhtar M. Feirtag J. Diez-Gonzalez F. (2011a). Reduction of *Escherichia coli* O157:H7 viability on hard surfaces by treatment with a bacteriophage mixture. Int. J. Food Microbiol. 145, 37–42. 10.1016/j.ijfoodmicro.2010.11.021 21145610

[B113] Viazis S. Akhtar M. Feirtag J. Diez-Gonzalez F. (2011b). Reduction of *Escherichia coli* O157:H7 viability on leafy green vegetables by treatment with a bacteriophage mixture and trans-cinnamaldehyde. Food Microbiol. 28, 149–157. 10.1016/j.fm.2010.09.009 21056787

[B114] Wang W. Singh S. Zeng D. L. King K. Nema S. (2007). Antibody structure, instability, and formulation. J. Pharm.Sci. 96, 1–26. 10.1002/jps.20727 16998873

[B115] Willford J. Goodridge L. D. (2008). An integrated assay for rapid detection of *Escherichia coli* O157H:7 on beef samples. Food Prot. Trends 28, 468–472.

[B116] Wysok B. Uradzinski J. (2009). *Campylobacter* spp.—a significant microbiological hazard in food. I. Characteristics of *Campylobacter* species, infection source, epidemiology. Pol. J. Vet. Sci. 12, 141–148. 19459452

[B117] Yamamoto Y. (2002). PCR in diagnosis of infection: detection of bacteria in cerebrospinal fluids. Clin. Diagn. Lab. Immunol. 9, 508–514. 10.1128/cdli.9.3.508-514.2002 11986253 PMC119969

[B118] Yang H. Wang D. B. Dong Q. H. Zhang Z. P. Cui Z. Q. Deng J. Y.. (2012). Existence of separate domains in lysin PlyG for recognizing *Bacillus anthracis* spores and vegetative cells. Antimicrob. Agents Chemother. 56, 5031–5039. 10.1128/AAC.00891-12 22802245 PMC3457386

[B119] Young R. Blasi U. (1995). Holins—Form and function in bacteriophage lysis. FEMS Microbiol. Rev 17, 191–205. 10.1111/j.1574-6976.1995.tb00202.x 7669346

[B120] Yu J. P. Zhang Y. Zhang Y. Li H. Yang H. Wei H. P. (2016). Sensitive and rapid detection of *Staphylococcus aureus* in milk via cell binding domain of lysin. Biosens. Bioelectron. 77, 366–371. 10.1016/j.bios.2015.09.058 26433070

[B121] Zhang H. Bao H. Billington C. Hudson J. A. Wang R. (2012). Isolation and lytic activity of the *Listeria* bacteriophage endolysin LysZ5 against *Listeria monocytogenes* in soya milk. Food Microbiol. 31, 133–136. 10.1016/j.fm.2012.01.005 22475951

[B122] Zhen Y. Mi T. Yu Z. (2008). Detection of Phaeocystis globosa using sandwich hybridization integrated with nuclease protection assay (NPA-SH). J. Environ. Sci. (China) 20, 1481–1486. 10.1016/S1001-0742(08)62553-X 19209636

